# Use of Indocyanine Green Fluorescent Imaging in the Assessment of a Tongue Flap After Lateral Hemiglossectomy

**DOI:** 10.7759/cureus.15248

**Published:** 2021-05-26

**Authors:** Lisandro Montorfano, Stephen J Bordes, Ryan Azarkhail, Mauricio Sarmiento Cobos, Michael Medina

**Affiliations:** 1 Surgery, Cleveland Clinic Florida, Weston, USA; 2 Surgery, Louisiana State University Health Sciences Center, New Orleans, USA; 3 Surgical Anatomy, Tulane University School of Medicine, New Orleans, USA; 4 Surgery, Ross University School of Medicine, Bridgetown, BRB; 5 Head and Neck Surgery, Cleveland Clinic Florida, Weston, USA

**Keywords:** icg angiography, indocyanine green, hemiglossectomy, tongue flap, squamous cell carcinoma (scc)

## Abstract

Indocyanine green (ICG) angiography is a real-time imaging modality that can be used to assess intraoperative tissue perfusion. ICG dye has proven to be feasible, safe, and cost-effective, especially for muscle flaps during complex reconstructions. To our knowledge, we discuss the first use of ICG angiography for the real-time assessment of a tongue flap following left lateral hemiglossectomy. ICG angiography showed excellent perfusion of the tongue and tongue flap, which subsequently led to an uncomplicated postoperative recovery.

## Introduction

Reconstruction after tongue cancer surgery can be challenging due to the complex anatomy and physiology of the tongue, depending on the location and extension of the tumor. When surgery is performed, adequate margins (>1 cm) are needed to decrease local recurrence [1]. Patients are often left with a cosmetic deformity. Reconstructions can be completed with local/regional flaps or free flaps. The goal is to achieve the best possible functional and oncologic outcomes. Various technologies have been developed to provide an objective assessment of flap perfusion, including indocyanine green (ICG) fluorescence. ICG is a helpful and safe tool for the assessment of flap perfusion as it is a relatively safe and nontoxic tricarbocyanine dye that allows for real-time visualization [2-4]. We discuss the use of ICG fluorescence for a tongue flap following a lateral hemiglossectomy.

## Case presentation

**Figure 1:**
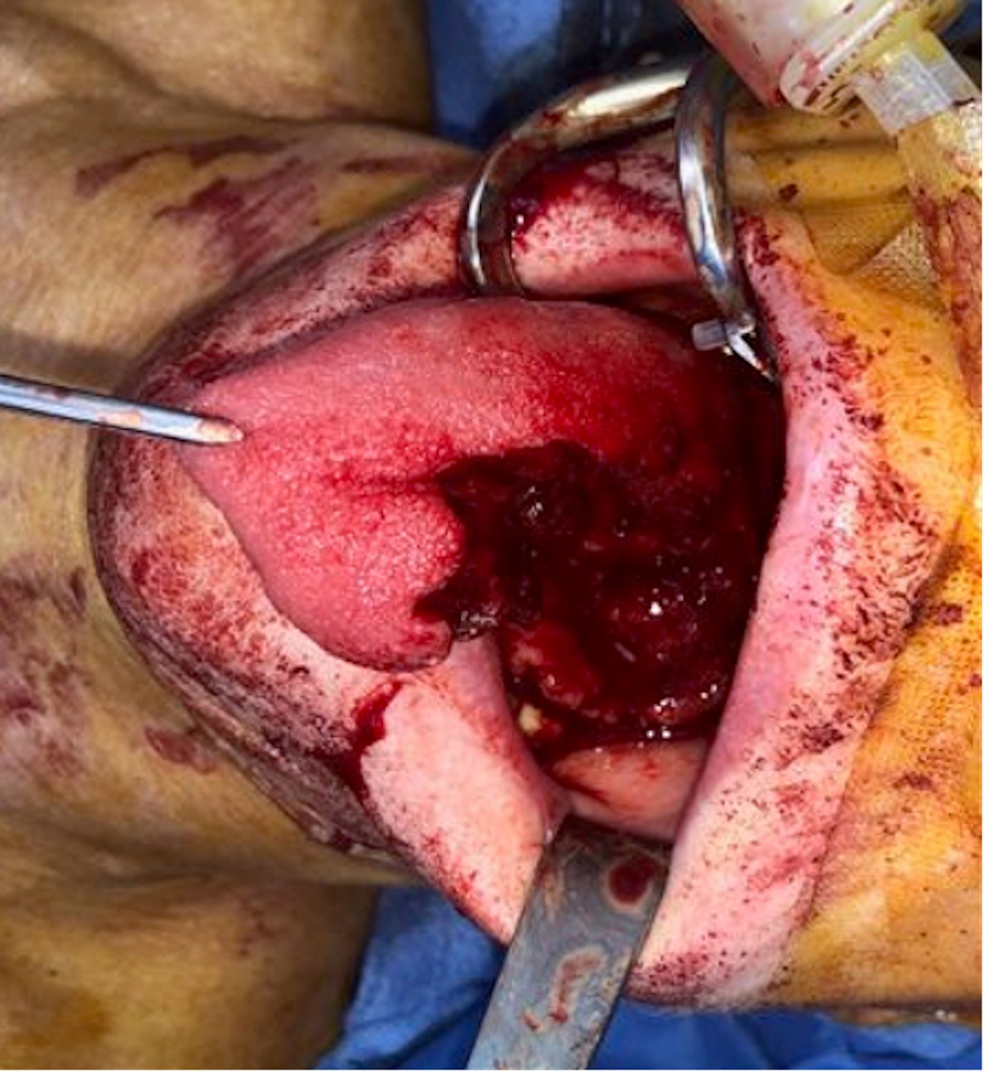
Left lateral hemiglossectomy.

A 51-year-old Caucasian woman, with a history of celiac disease and squamous cell carcinoma of the tongue, came to our institution for a left lateral hemiglossectomy with reconstruction. The patient was brought to the operating room and placed on the table in the supine position. General anesthesia was administered, and the patient was intubated. The patient’s face and neck were then prepped and draped in a sterile fashion. A side gag was placed opposite the tumor. A penetrating towel clamp was used to retract the tongue anteriorly and away from the tumor. We marked a 1 cm margin around the primary lesion. Using a CO_2_ laser system, we performed a left lateral hemiglossectomy (Figure 1). Hemostasis was attained with cautery (bipolar and monopolar) and 3-0 vicryl suture ligatures for branches of the lingual artery. Once the primary lesion was removed, we resected peripheral mucosal margins and sent them to the pathology department for the frozen section. The frozen sections showed no signs of cancer.

**Figure 2:**
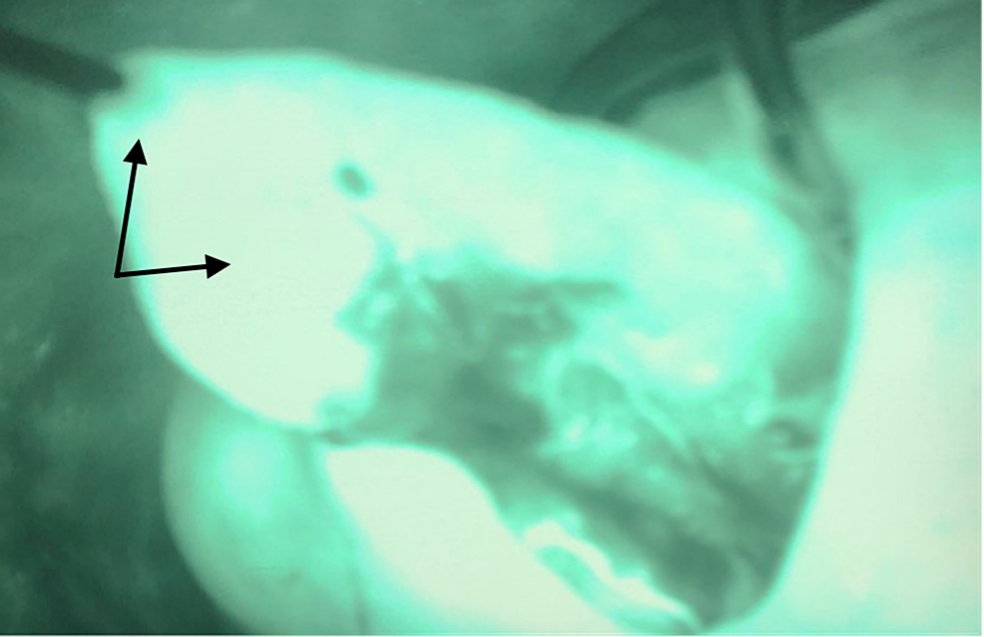
ICG angiography showing perfusion of the tongue following left lateral hemiglossectomy. Successful perfusion is noted by fluorescent green emission. Arrows point to both the tip of the tongue and the left anterior tongue. ICG: indocyanine green

**Figure 3:**
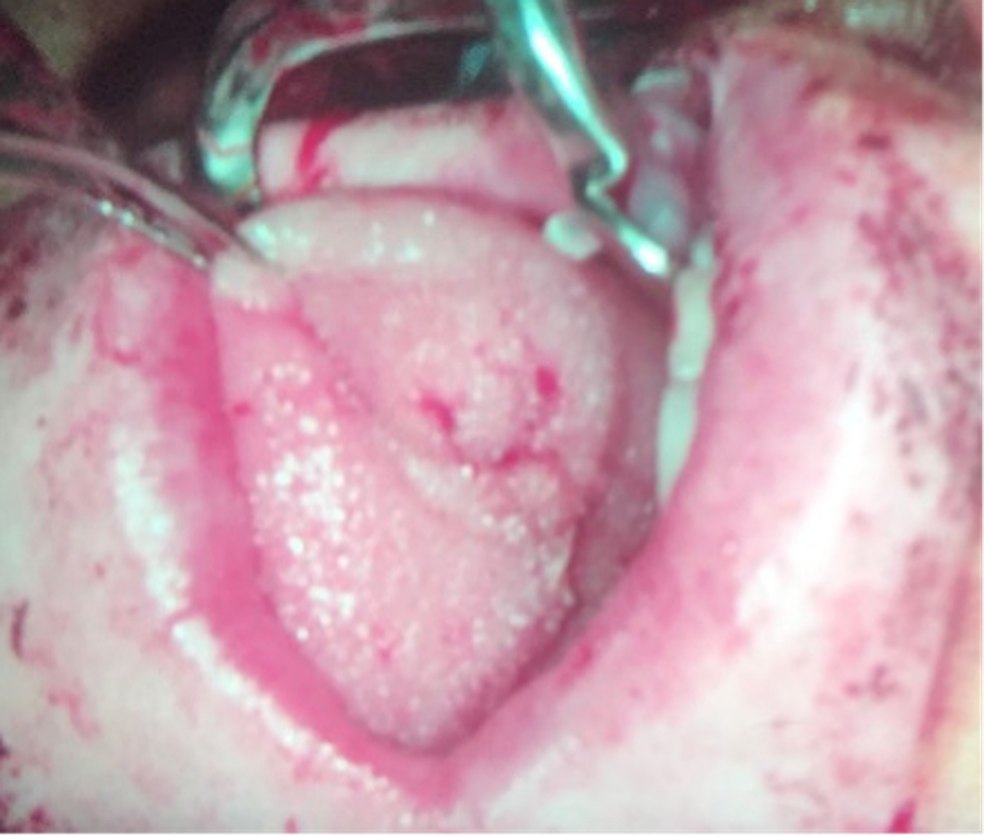
Tongue flap reconstruction.

**Figure 4:**
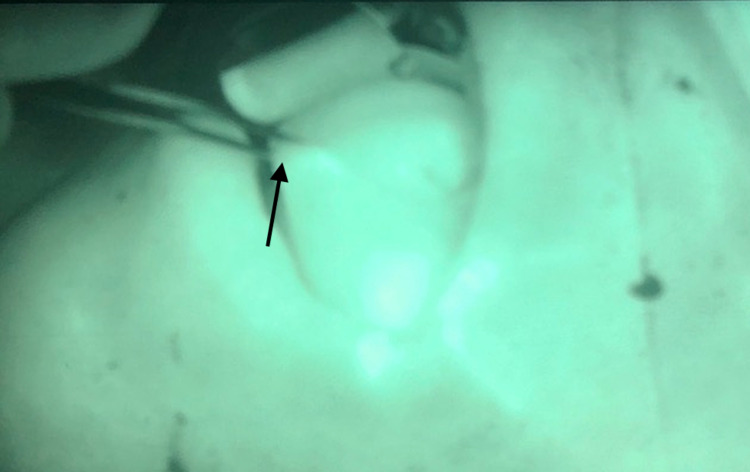
Tongue flap perfusion with ICG. Arrow points to the tip of the tongue. ICG: indocyanine green

Subsequently, we used ICG fluorescence to determine the viability of the left anterior tongue due to concerns for perfusion, especially at the tip, as an extensive resection across the midline was performed. During the procedure, 1 cc of ICG was administered intravenously followed by a 10 cc flush of normal saline. The tongue was assessed immediately after injection using a Stryker SPY-PHI (Stryker Corporation, Kalamazoo, MI) portable handheld imaging system. ICG fluorescence showed good perfusion of the tongue (Figure 2). We then rotated the tongue posteriorly to cover the defect. This local flap was sutured in place using 3-0 vicryl interrupted sutures (Figure 3). We then used ICG angiography to reassess the flap. The reconstructed tongue had good perfusion (Figure 4). We subsequently performed a bilateral neck dissection. The wounds were irrigated copiously with water and saline. Two 19-French Blake round drains were placed in the neck and secured with 2-0 nylon sutures. Closure of the skin was performed in layers with 3-0 vicryl sutures for the platysma layer and staples for the skin. The patient was awakened and transferred to the recovery room in stable condition. Two weeks later, the patient was seen in the office. No complications from the reconstruction were reported.

## Discussion

ICG angiography has revolutionized surgeons’ ability to assess tissue perfusion in the operating room. First introduced in the 1950s, ICG is a unique tricarbocyanine dye that allows real-time targeted anatomical visualization due to its high level of contrast against the background, innate ability to be taken up in small concentrations, rapid excretion into the bile, ease of use for prospective clinicians and researchers, low toxicity potential, and affordability [5-7].

Due to its amphiphilic characteristics, ICG has a nonspecific affinity for plasma proteins and blood lipoproteins [8]. The resulting interactions and competition for binding causes aggregation, which subsequently permits ICG to affect the emission spectrum and results in its fluorescent capabilities [8]. ICG fluorescence is best observed within a few seconds of intravenous injection between the wavelengths of 820 and 830 nm on the emission spectrum in vitro [8]. To achieve a plasma concentration of 100 mg/mL, dosages are calculated at 0.5 mg/kg body weight [9]. Furthermore, ICG has demonstrated a rapid clearance (half-life of 3-5 minutes) from the body via first-order kinetics in the form of nonconjugated bile [2,5]. ICG also has a very low toxicity profile, with reported cases of 1/40,000 [6,7,9].

ICG stands as a frontier for further surgical utilization, research, and clinical usage. The application of this imaging modality first gained traction through its vascular applications in ophthalmology, cardiology, and neurosurgery [10]. As continued research and case studies reveal, ICG has proven to be versatile across many specialties. Investigations have highlighted the potential for ICG to be used in postoperative wound care, lymph node biopsy, cancer treatment and detection, evaluation of liver function, and plastic surgery reconstruction [6,7,10].

Vascular patency and quality blood flow are vital for the long-term success and sustainability of any flap. This principle holds particularly true for muscle-only flaps, which exhibit the lowest salvage rate of all flap types [11]. Our case demonstrates the utility of ICG angiography for the real-time intraoperative vascular assessment of a rotational tongue flap following lateral hemiglossectomy. While several studies have described flap perfusion assessment with ICG, our study is the first to highlight the application of ICG angiography exclusively for tongue reconstruction. Upon the initial phase of reconstruction, ICG was used to evaluate blood flow of the left anterior tongue following left lateral hemiglossectomy to rule out any potential vascular compromise. ICG was then used to reassess the vascularization of the tongue following reconstruction. Our patient experienced no postoperative complications.

Our study not only asserted the effectiveness of ICG for tongue flap reconstruction but also revealed its potential superiority to pinprick testing. Although simple, quick, and conventional, pinprick testing is largely dependent on physician experience due to the subjective nature of the results [12]. Additionally, bruising at the site can develop in patients who have undergone repeated testing, jeopardizing the reliability of the examination [12]. In the setting of free-flap vascular complications in patients who have undergone microvascular reconstruction, ICG was able to identify intraoperative and postoperative complications at an earlier rate than clinical examination alone; however, data on locoregional flaps is limited [13]. Successful salvage is fundamentally reliant on early detection of potential complications, such as those related to hypoperfusion of tissues. [14]. Our case highlights the feasibility, safety, and efficacy of ICG angiography in determining short-term and long-term flap viability.

As ICG angiography continues to grow in use and popularity, future investigations are necessary to accrue clinical outcome data with greater study power and compare ICG angiography to other intraoperative assessment modalities for locoregional tongue flaps.

## Conclusions

The use of ICG angiography for the real-time assessment of blood flow is a valuable and feasible tool for the intraoperative assessment of perfusion in extended hemiglossectomies with local flap reconstruction. It may be especially beneficial in cases involving complex reconstructions, and when regional organ hypoperfusion is suspected.

## References

[1] Hicks WL Jr, North JH Jr, Loree TR (1998). Surgery as a single modality therapy for squamous cell carcinoma of the oral tongue. Am J Otolaryngol.

[2] Abdelwahab M, Kandathil CK, Most SP, Spataro EA (2019). Utility of indocyanine green angiography to identify clinical factors associated with perfusion of paramedian forehead flaps during nasal reconstruction surgery. JAMA Facial Plast Surg.

[3] Gurtner GC, Jones GE, Neligan PC, Newman MI, Phillips BT, Sacks JM, Zenn MR (2013). Intraoperative laser angiography using the SPY system: review of the literature and recommendations for use. Ann Surg Innov Res.

[4] Lee BT, Matsui A, Hutteman M, Lin SJ, Winer JH, Laurence RG, Frangioni JV (2010). Intraoperative near-infrared fluorescence imaging in perforator flap reconstruction: current research and early clinical experience. J Reconstr Microsurg.

[5] Alander JT, Kaartinen I, Laakso A (2012). A review of indocyanine green fluorescent imaging in surgery. Int J Biomed Imaging.

[6] Montorfano L, Bordes SJ, Sarmiento Cobos M, Garcia Lopez EA, Medina M (2021). Use of indocyanine green angiography for real-time assessment of a sternocleidomastoid muscle flap during complex facial reconstruction. Cureus.

[7] Ferri F, Montorfano L, Bordes SJ, Forleiter C, Newman MI (2021). Near-infrared fluorescence imaging for sentinel lymph node identification in melanoma surgery. Cureus.

[8] Desmettre T, Devoisselle JM, Mordon S (2000). Fluorescence properties and metabolic features of indocyanine green (ICG) as related to angiography. Surv Ophthalmol.

[9] De Gasperi A, Mazza E, Prosperi M (2016). Indocyanine green kinetics to assess liver function: ready for a clinical dynamic assessment in major liver surgery?. World J Hepatol.

[10] Reinhart MB, Huntington CR, Blair LJ, Heniford BT, Augenstein VA (2016). Indocyanine green: historical context, current applications, and future considerations. Surg Innov.

[11] Chang EI, Zhang H, Liu J, Yu P, Skoracki RJ, Hanasono MM (2016). Analysis of risk factors for flap loss and salvage in free flap head and neck reconstruction. Head Neck.

[12] Nagata T, Masumoto K, Uchiyama Y, Watanabe Y, Azuma R, Morimoto Y, Katou F (2014). Improved technique for evaluating oral free flaps by pinprick testing assisted by indocyanine green near-infrared fluorescence angiography. J Craniomaxillofac Surg.

[13] Hitier M, Cracowski JL, Hamou C, Righini C, Bettega G (2016). Indocyanine green fluorescence angiography for free flap monitoring: a pilot study. J Craniomaxillofac Surg.

[14] Betz CS, Zhorzel S, Schachenmayr H (2009). Endoscopic measurements of free-flap perfusion in the head and neck region using red-excited indocyanine green: preliminary results. J Plast Reconstr Aesthet Surg.

